# Kidney Dysfunction Impact on White Matter Hyperintensity Volume in Neurologically Healthy Adults

**DOI:** 10.1038/s41598-019-45109-y

**Published:** 2019-06-13

**Authors:** Sang Hyuck Kim, Jae Moon Yun, Su-Min Jeong, Shinhye Kim, Tae Gon Yoo, Ji Eun Lee, Jae-Sung Lim, Han-Yeoung Jeong, Ki-Woong Nam, Hyung-Min Kwon, Jin-Ho Park

**Affiliations:** 1 Department of Family Medicine, Bumin Hospital, Seoul, Republic of Korea; 2 Department of Family Medicine, Seoul National University Hospital, Seoul, Republic of Korea; 3 Department of Family Medicine, Gangnam Severance Hospital, Seoul, Republic of Korea; 4 Department of Neurology, Hallym University Sacred Heart Hospital, Anyang, Republic of Korea; 5 Department of Neurology, Seoul National University-Seoul Municipal Government Boramae Medical Center, Seoul, Republic of Korea; 6 Department of Neurology, Seoul National University College of Medicine, Seoul, Republic of Korea; 7 Department of Family Medicine, Seoul National University College of Medicine, Seoul, Republic of Korea

**Keywords:** Stroke, White matter disease

## Abstract

The detrimental outcomes of white matter hyperintensity (WMH) are known to be proportional to WMH volume. This study aimed to evaluate the association between kidney dysfunction and white matter hyperintensity (WMH) volume. A total of 2,203 subjects who underwent brain magnetic resonance imaging (MRI) as part of a screening health check-up was included in this study. WMH was defined as hyperintensity signals without cavity formation in the white matter on fluid-attenuated inversion recovery images. WMH volume was measured quantitatively, and data were normalized by square root transformation prior to analysis. Mean age of the subjects was 56.9 years and mean WMH volume was 2.7 cm^3^. Mean estimated glomerular filtration rate (eGFR) level was 78.0 ml/min/1.73 m^2^, and 172 subjects (7.8%) were diagnosed with moderate-to-severe kidney dysfunction (eGFR < 60). Mean Urine albumin-to-creatinine ratio (UACR) was 0.02, and 166 subjects showed significant albuminuria (UACR ≥ 0.03). Multivariate analyses showed that each of UACR, significant albuminuria, and moderate-to-severe kidney dysfunction was significantly associated with increased WMH volume (all *p* < 0.05). When we considered significant albuminuria and moderate-to-severe kidney dysfunction simultaneously, subjects with both significant albuminuria and moderate-to-severe kidney dysfunction had more than twice the WMH volume as did those in the other groups (all *p* < 0.05). Kidney dysfunction, defined by albuminuria and eGFR, was independently associated with WMH volume. Risk factors related to WMH and its detrimental outcomes should be strictly modified in subjects with kidney dysfunction, especially in those with both albuminuria and a reduced eGFR.

## Introduction

White matter hyperintensity (WMH) is a frequently encountered abnormal finding in brain magnetic resonance imaging (MRI) studies. This anomaly is more frequently observed in the elderly population and is regarded as a non-specific finding associated with the aging process^1,2^. However, mounting clinical evidence supports the fact that an advanced degree of WMH is associated with several physical dysfunctions and clinically detrimental outcomes^2–4^. Research studies have shown that WMH is associated with cognitive impairment, gait disturbance, mood change, functional decline, and urinary incontinence^2^. In addition, WMH increases the risk of stroke and stroke-related complications, including vascular dementia, and bleeding after thrombolysis^2–4^. Consequently, due to these clinical implications, WMH is regarded as a potential surrogate marker of overt stroke^3^.

Since the microvascular structures of both the kidney and the brain are similar, it has been suggested that markers for chronic kidney disease could be predictors of cerebral vascular diseases including WMH^5,6^. Considering that the detrimental outcomes of WMH are known to be proportional to WMH volume^2^, quantitative volumetric assessment is critical in evaluating the association between markers for kidney dysfunction and WMH. In addition to the shortage of studies targeting the association between kidney dysfunction and WMH volume, the volumetric complexity and characteristics of WMH itself can cause methodological limitations, such as using the WMH grade instead of volume itself^7,8^, and the inappropriate treatment of a zero value for WMH volume during logarithmic transformation^9,10^.

This study aimed to assess the association between kidney dysfunction, as defined by urine albumin-to-creatinine ratio (UACR), estimated glomerular filtration rate (eGFR), and quantitative WMH volume, as measured by a semi-automated technique.

## Methods

### Subjects and variables

This study included subjects aged 40 years or older who, at their own expense, underwent routine health check-ups including brain MRI at Seoul National University Hospital Health Promotion Center between January 2006 and December 2013. Upon their initial visit, subjects were asked to complete detailed questionnaires regarding their sociodemographic background, lifestyle, and medical history. Subjects then consulted with a trained family physician with regard to their past medical histories and current medications. In terms of smoking habit, subjects were categorized as (1) non- or former smoker, or (2) current smoker. We also identified subjects who were taking anti-hypertensive, anti-diabetic, anti-dyslipidemic, or anti-coagulation/anti-platelet drugs. The screening program included anthropometric assessment, blood pressure, electrocardiogram, blood sampling for metabolic and biochemical traits after overnight fasting, urinary tests, and brain MRI. Hypertension was defined as when subjects were taking antihypertensive drugs or when systolic blood pressure (SBP) ≥ 140 mmHg, or when diastolic blood pressure (DBP) ≥ 90 mmHg. Diabetes was defined when subjects were taking antidiabetic drugs or had a fasting blood glucose level ≥126 mg/dL or an HbA1c ≥6.5%. Dyslipidemia was defined when subjects were taking lipid lowering drugs or when their total cholesterol level was ≥240 mg/dL. The detailed MRI protocol is described elsewhere^11^.

A total of 3,131 subjects, aged 40 years or more, underwent brain MRI as part of their routine health screening check-ups. Of these, 925 subjects were excluded due to missing urinary albumin and creatinine levels. We also excluded 3 subjects who had a previous history of stroke. Finally, a total of 2,203 subjects were included in our analysis.

This study was approved by the institutional review board at Seoul National University Hospital. (IRB No. H-1604-072-754). Informed consent was exempted by the IRB due to anonymous information collection and retrospective study design. All methods were performed in accordance with relevant guidelines and regulations.

### Assessment of the volume of white matter hyperintensity

Hyperintensity signals without cavity formation (in other words, signal which differed from that of the cerebrospinal fluid)that appeared in a variety of different sizes in the white matter upon fluid-attenuated inversion recovery images from brain MRI were defined as WMH.^1^Lesions in the subcortical grey matter or brainstem were not included in this category. The WMH volume (cm^3^) of each subject was calculated using a semi-automated technique using MRIcro software (http://www.mricro.com). Semi-automated volumetric WMH measurements are more objective and reliable than qualitative scales. In addition, this method overcomes the shortcoming of visual ratings, such as ceiling effects. Scans were converted from DICOM to Analyze format, for computer-assisted determination of WMH volume. For each subject, the signal intensity threshold for WMH was manually edited and used to create a region-of-interest map of supratentorial WMHs. All measurements were performed independently by researchers who were blinded to clinical data.

### Kidney function assessment

UACR was determined from the quantitative measures obtained for urinary albumin and creatinine excretion and then used as a parameter for kidney dysfunction. eGFR was calculated from the Modification of Diet in Renal Disease (MDRD) formula (GFR = 175 × serum creatinine^−1.154^ × age^−0.203^ × 0.742 [for females])^12^. Kidney function was categorized as normal (≥90) or mild dysfunction (60 to 89.9), moderate dysfunction (30 to 59.9), or severe dysfunction (<30 mL/min/1.73 m^2^)^13^.

### Statistical analysis

WMH volume data were extremely skewed to the left side. Consequently, these data needed to be transformed prior to analysis. Due to the large number of subjects with a WMH volume of zero (n = 537, 22.4%), we opted to treat WMH data by square root transformation $(\sqrt{{\rm{WMH}}\,{\rm{volume}}})$.

At first, we performed univariate linear regression analyses to investigate the association between WMH volume and each of the baseline characteristics. Considering previous studies, and the established cardiovascular disease risk factors^7–10,14,15^, the following variables were included as cofactors for multivariate analyses: age, sex smoking, body mass index, hypertension, diabetes, dyslipidemia, and the use of anti-coagulation or anti-platelet drugs. Multivariate linear regression analysis was used to assess the association between UACR level and WMH volume. The natural logarithm of UACR data was used as an independent variable. Next, we used the same multivariate analysis to investigate categorical variables of kidney dysfunction. In model I, kidney function was categorized according to the level of albuminuria. Significant albuminuria was defined as when UACR was ≥0.03^16^. In model II, categorization of kidney function according to eGFR level was used as a predictor variable. As only 3 (0.1%) had an eGFR < 30 ml/min/1.73 m^2^, we combined subjects with an eGFR < 30 and those with an eGFR of 30–59.9 into the eGFR < 60 category. In model III, a combination of albuminuria and eGFR data was considered simultaneously. Kidney function was categorized as follows: 1) no significant albuminuria and an eGFR ≥ 60 (reference group); 2) an eGFR < 60 only (without proteinuria); 3) significant albuminuria only (eGFR ≥ 60); and 4) significant albuminuria and an eGFR < 60. To present volumetric differences across the kidney function categories in model III, we calculated adjusted means for the square root of WMH volumes. The calculated adjusted means, and their confidence intervals, for the square root of WMH volume were then re-squared and Tukey’s post hoc analysis performed.

All statistical analyses were conducted using STATA software version 14.1 (StataCorp., TX) and *p* values less than 0.05 were considered statistically significant.

## Results

### Baseline characteristics and univariate analysis

The distribution of baseline characteristics is shown in Table 1. Mean WMH volume was 2.7 ± 6.0 cm^3^ and mean age was 56.9 ± 8.3 years. In total, 1,215 (55.2%) subjects were male. Mean systolic and diastolic blood pressures were 126.6 ± 15.8and 76.3 ± 10.6 mmHg, respectively. Mean serum creatinine level was within normal range (0.92 mg/dL) and mean eGFR was 78.0 ± 14.8 ml/min/1.73 m^2^. Mean UACR was 0.02 ± 0.10and 166 subjects (7.5%) showed significant albuminuria.

**Table 1:** Baseline characteristics of study subjects (*N* = 2,203)

White Matter Hyperintensity Volume - cm^3^	2.7	±6.0
Age (year)	56.9	±8.3
Male, n (%)	1,215	(55.2)
Current Smoker, n (%)	410	(18.6)
Taking Anti-hypertensive Drugs, n. (%)	551	(25.0)
Taking Anti-diabetic Drugs, n. (%)	154	(7.0)
Taking Anti-dyslipidemic Drugs, n (%)	177	(8.0)
Taking Anti-coagulation or Anti-platelet Drugs^*^, n (%)	224	(10.2)
Body Mass Index - kg/m^2^	24.1	±3.0
Systolic Blood Pressure - mmHg	126.6	±15.8
Diastolic Blood Pressure - mmHg	76.3	±10.6
Creatinine - mg/dL	0.92	±0.21
eGFR^†^ - ml/min/1.73 m^2^	78.0	±14.8
≥90 ml/min/1.73 m^2^, n (%)	421	(19.1)
60–89.9 ml/min/1.73 m^2^, n (%)	1610	(73.1)
30–59.9 ml/min/1.73 m^2^, n (%^)^	169	(7.7)
<30 ml/min/1.73 m^2^, n (%)	3	(0.1)
Urine Albumin-to-Creatinine Ratio	0.02	±0.10
<0.03	2,037	(92.5)
≥0.03	166	(7.5)
Total Cholesterol - mg/dL	200.7	±35.6
Low Density Lipoprotein - mg/dL	126.3	±34.3
High Density Lipoprotein - mg/dL	54.5	±13.7
Triglyceride - mg/dL	119.6	±70.0
Hemoglobin A1c	5.92	±0.78
Hypertension^‡^, n (%)	859	(39.0)
Diabetes^§^, n (%)	330	(15.0)
Dyslipidemia^¶^, n (%)	457	(20.7)

eGFR, estimated glomerular filtration rate.

Values are shown as means ± standard deviation.

^*^Subjects taking aspirin, plavix, warfarin, or other antiplatelet drugs.

^†^Calculated using the Modification of Diet in Renal Disease formula.

^‡^Subjects taking antihypertensive drugs or with a SBP ≥ 140 mmHg, or a DBP ≥90 mmHg.

^§^Subjects taking antidiabetic drugs or with a fasting blood glucose ≥ 126 mg/dL or a HbA1c ≥6.5%.

^¶^Subjects taking lipid lowering drugs or with a total cholesterol level ≥ 240 mg/dL.

In our univariate analyses, we found that the following factors were significantly associated with a change in WMH volume: age, smoking habit, taking anti-hypertensive, anti-diabetic, anti-dyslipidemic drugs, anti-coagulation/anti-platelet drugs, systolic and diastolic blood pressure, eGFR, UACR, total cholesterol, low density lipoprotein, serum hemoglobin A1c level, hypertension, and diabetes (Table 2).

**Table 2:** Univariate Linear Regression Between White Matter Hyperintensity Volume and Potential Risk Factors.

	β	(95% CI)	*P*
Age	0.060	(0.055 to 0.065)	<0.001
Male	0.038	(−0.055 to 0.131)	0.424
Current Smoker	−0.196	(−0.315 to −0.077)	0.001
Taking Anti-hypertensive Drugs	0.402	(0.296 to 0.507)	<0.001
Taking Anti-diabetic Drugs	0.403	(0.222 to 0.584)	<0.001
Taking Anti-dyslipidemic Drugs	0.174	(0.003 to 0.344)	0.046
Taking Anti-coagulation or Anti-platelet Drugs^*^	0.309	(0.156 to 0.462)	<0.001
Body Mass Index	−0.010	(−0.026 to 0.005)	0.193
Systolic Blood Pressure	0.013	(0.010 to 0.016)	<0.001
Diastolic Blood Pressure	0.011	(0.006 to 0.015)	<0.001
Creatinine	0.409	(0.184 to 0.634)	<0.001
eGFR^†^ - ml/min/1.73 m^2^	−0.007	(−0.010 to −0.004)	<0.001
≥90 ml/min/1.73 m^2^ - no. (%)			*reference*
60–89.9 ml/min/1.73 m^2^ - no. (%)	0.043	(−0.075 to 0.161)	0.471
30–59.9 ml/min/1.73 m^2^ - no. (%)	0.499	(0.303 to 0.695)	<0.001
<30 ml/min/1.73 m^2^ - no. (%)	0.307	(1.825 to 4.320)	<0.001
Urine Albumin/Creatinine Ratio^‡^	0.231	(0.185 to 0.278)	<0.001
<0.03			*reference*
≥0.03	0.463	(0.288 to 0.637)	<0.001
Total Cholesterol - mg/dL	−0.001	(−0.003 to 0.000)	0.047
Low Density Lipoprotein - mg/dL	−0.001	(−0.003 to 0.000)	0.041
High Density Lipoprotein - mg/dL	−0.001	(−0.004 to 0.002)	0.558
Triglyceride - mg/dL	0.000	(−0.000 to 0.001)	0.297
Hemoglobin A1c - %	0.150	(0.091 to 0.210)	<0.001
Hypertension^§^	0.450	(0.357 to 0.543)	<0.001
Diabetes^¶^	0.406	(0.278 to 0.535)	<0.001
Dyslipidemia^**^	0.042	(−0.072 to 0.156)	0.472

CI, confidence interval; eGFR, estimated glomerular filtration rate.

^*^Those who were taking aspirin, clopidogrel, warfarin, or other antiplatelet drugs.

^†^Calculated using the Modification of Diet in Renal Disease formula.

^‡^Natural logarithm of urine microalbumin/creatinine ratio.

^§^Those who were taking antihypertensive drugs or SBP ≥ 140 mmHg, or DBP ≥ 90 mmHg.

^¶^Those who were taking antidiabetic drugs or fasting blood glucose ≥ 126 mg/dL or Hb A1c ≥ 6.5%.

^**^Those who were taking lipid lowering drugs or total cholesterol ≥ 240 mg/dL.

### Association between kidney dysfunction and WMH volume

**Figure 1:**
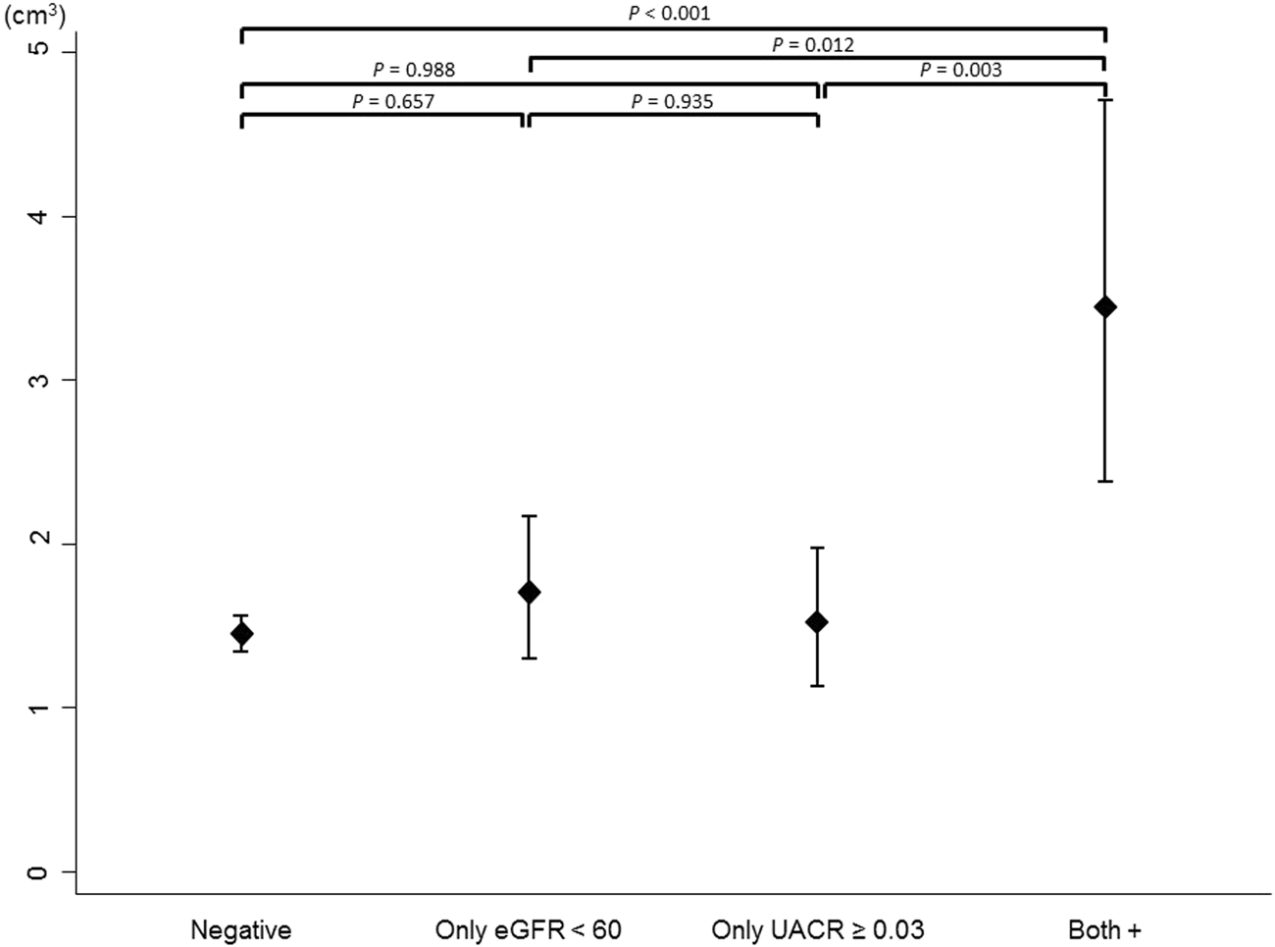
Adjusted means of white matter hyperintensity volume by kidney dysfunction groups. Adjusted means of WMH volume are shown with confidence intervals (adjusted for age, sex, smoking, body mass index, hypertension, diabetes, dyslipidemia, and the use of anti-coagulation or anti-platelet drugs). The calculated adjusted means, and their confidence intervals for the square root of white matter hyperintensity volume, were re-squared and Tukey’s post-hoc analysis was used to calculate *p* values. (eGFR, estimated glomerular filtration rate; UACR, urine albumin-to-creatinine ratio).

In our multivariate regression analysis, UACR was independently associated with a change in WMH volume (β = 0.113; *p* < 0.001) after adjustment for the above-mentioned demographic, lifestyle, and clinical factors (Table 3). Subjects with significant albuminuria (β = 0.163; *p* = 0.045) or moderate-to-severe kidney dysfunction (β = 0.219; *p* = 0.005) had a higher WMH volume. The association between eGFR < 60 only (without albuminuria) or significant albuminuria only (with normal eGFR) and WMH volume was not statistically significant. However, subjects with both significant albuminuria and an eGFR < 60 had a significantly higher WMH volume (β = 0.652; *p* < 0.001) (Table 4). The calculated WMH volume in subjects with both significant albuminuria and an eGFR < 60 (volume = 3.45 cm^3^; confidence interval (CI), 2.38 to 4.71) had more than twice the volume of WMH as did those in the other groups(for the normal group: volume = 1.45, CI, 1.35 to 1.56; for the eGFR < 60 only group: volume = 1.71 cm^3^,CI, 1.30 to 2.17;for the significant albuminuria only group: volume = 1.52, CI, 1.13 to 1.98) (Fig. 1).

**Table 3:** Multivariate Linear Regression between White Matter Hyperintensity Volume and Potential Risk Factors.

	Changes in Square Root of WMH Volume (95% CI)		P*
Urine Albumin-to-Creatinine Ratio^†^	0.113	(0.068 to 0.158)	<0.001
Age	0.055	(0.050 to 0.061)	<0.001
Sex (Male)	0.061	(−0.028 to 0.151)	0.180
Current Smoking	0.006	(−0.108 to 0.120)	0.919
BMI	−0.013	(−0.027 to 0.001)	0.071
Hypertension	0.253	(0.164 to 0.642)	<0.001
Diabetes	0.063	(−0.059 to 0.185)	0.314
Dyslipidemia	−0.063	(−0.165 to 0.039)	0.228
Taking Anticoagulation or Anti-platelet Drugs^‡^	−0.054	(−0.193 to 0.086)	0.450

WMH, white matter hyperintensity; CI, confidence interval; eGFR, estimated glomerular filtration rate.

^*^P values were calculated by multivariate linear regression analysis adjusted for age, sex, smoking, body mass index, hypertension, diabetes, dyslipidemia, and the use of anti-coagulation or anti-platelet drugs.

^†^Natural logarithm of urine albumin-to-creatinine ratio.

^‡^Subjects taking aspirin, plavix, warfarin, or other antiplatelet drugs.

**Table 4:** Changes in White Matter Hyperintensity Volume according to Kidney Functions Groups.

	Changes in Square Root of WMH Volume (95% CI)		*P* ^*^
Model I (UACR)			
<0.03	Reference		
≥0.03	0.163	(0.004 to 0.322)	0.045
Model II (eGFR)			
≥60	Reference		
<60	0.219	(0.065 to 0.374)	0.005
Model III (UACR/eGFR)			
Negative (*N* = 1,903)	Reference		
Only eGFR < 60 (*N* = 134)	0.102	(−0.071 to 0.275)	0.250
Only UACR ≥0.03 (*N* = 128)	0.030	(−0.149 to 0.209)	0.745
Both+ (*N* = 38)	0.652	(0.336 to 0.969)	<0.001

WMH, white matter hyperintensity; UACR, urine albumin-to-creatinine ratio, CI, confidence interval; eGFR, estimated glomerular filtration rate.

*P values were calculated by multivariate linear regression analysis adjusted for age, sex, smoking, body mass index, hypertension, diabetes, dyslipidemia, and the use of anti-coagulation or anti-platelet drugs.

## Discussion

In this study, we investigated the association between kidney dysfunction, as defined by UACR and eGFR, and WMH volume in neurologically healthy adults encompassing a wide age range of subjects by using quantitative WMH volume data after adjustments for potential demographic, lifestyle, and clinical risk factors. We found that the presence and degree of kidney dysfunction, as defined by albuminuria and eGFR level, was independently associated with WMH volume.

Cerebral small vessel diseases including WMH are known to result from endothelial impairment and related vascular dysfunction^17^. In addition, subjects with kidney dysfunction are also known to be prone to such endothelial damages and thromboembolic events^6,18–20^. As both WMH and kidney dysfunction share the pathophysiological features^21^, the association between the impairments of these two end-organs was of great interest. Although a limited number of previous studies have dealt with kidney dysfunction and the volumetric degree of WMH, they had a variety of methodological limitations including relatively small sample size^7,9^, deviated age groups^7,9^, the use of simple grading methods for WMH volume such as measuring volume in a non-quantitative manner^7,8^, and bias-related log-transformation of WMH volume resulting in a large number of ‘zero’ values^9,10^.

The present study involved a large sample size and covered various age-groups. In total, 449 subjects (20.4%) were in their 40 s, while 960 (43.6%) were in their 50 s; none had any overt history of stroke. Furthermore, we measured WMH volume in a direct manner using a semi-automated method, which supports the reliability of the volumetric association between kidney dysfunction and WMH. Moreover, as we normalized the distribution of WMH volume using a square root transformation, possible bias related with ‘zero WMH’ value was avoided. These characteristics of the present study strongly support the generalizability of our results.

Consistent with previous studies, we found that kidney dysfunction was independently associated with WMH volume regardless of various confounding factors^7–10^. In addition, decreased eGFR and UACR seem to be synergistically associated with WMH volume. Conversely, the WMH volume was not significantly increased in subjects with isolated decreased eGFR or isolated UACR abnormality. The results could be explained based on transient conditions. Unfortunately, our study was based on tests performed at a single time point. Transient albuminuria can be shown in the normal population^22^. Especially, the overnight fasting condition in our study may have caused transient albuminuria or decreased eGFR by dehydration. However, in the cases with both albuminuria and decreased eGFR, we can assume the high possibility of it resulting from chronic kidney dysfunction or a more severe status. Since this chronic condition may affect the brain, it is understandable that subjects with both albuminuria and decreased eGFR had significantly larger WMH volumes^22^.

Systolic blood pressure and the use of antihypertensive medications were also significantly associated with WMH volume. As WMH is associated with overt stroke and related complications^2–4^, the reduction of blood pressure in subjects with hypertension might be effective in both kidney protection and stoke prevention^23^. However, although the association between kidney dysfunction and WMH volume was independent of systolic blood pressure or antihypertensive medication, it is still unclear whether kidney dysfunction and WMH volume are independent concurrent impairments caused by hypertension or whether kidney dysfunction is an independent risk factor of WMH volume.

One notable limitation of the present study is its cross-sectional design that could not guarantee a causal relationship between risk factors and WMH volume. To partially address this limitation, we included various potential co-factors in our statistical analysis. However, the observational study design still made it impossible to assert a causal relationship. Longitudinal studies are now needed to address this issue. Another limitation is the possible selection bias caused by income status or health demands. The cost of the screening program including brain MRI is between $1,000 to $4,000 and is not covered by insurance. The participants of this study may have higher incomes or higher health demands that can justify the cost. However, we suggest that there was little difference between our participants and the general population since most participants had neither symptoms nor prior diagnosis of brain lesions, and the prevalence of major comorbid conditions (hypertension, diabetes mellitus, and dyslipidemia) was similar to that of the general population in South Korea^24^. Similarly, participant selection bias for UACR may remain. Usually, more tests could be performed in the higher risk group. Indeed, there were statistical differences between subjects with and without UACR (Supplementary Table 1). However, subjects with UACR showed higher risk in several factors, such as systolic blood pressure, GFR, and hemoglobin A1c, and lower risk in other factors, such as age and fasting blood glucose level. We assume that the overall risk difference would not be large between both groups. Finally, kidney damage is defined by persistent albuminuria, while our study are limited to a single test. Future studies including repeated urine tests will be needed.

In conclusion, kidney dysfunction, as defined by albuminuria and eGFR, were independently associated with WMH volume in a neurologically healthy population of subjects undergoing routine health screening. Risk factors related to WMH and its detrimental outcomes should therefore be strictly modified in subjects with kidney dysfunction.

## Supplementary information

Baseline characteristics of Included and Excluded subjects based on the Urine Albumin-to-Creatinine Ratio

## References

[1] Wardlaw JM (2013). Neuroimaging standards for research into small vessel disease and its contribution to ageing and neurodegeneration. Lancet Neurol.

[2] Pantoni L (2008). Leukoaraiosis: from an ancient term to an actual marker of poor prognosis. Stroke.

[3] Inzitari D (2003). Leukoaraiosis: an independent risk factor for stroke?. Stroke.

[4] Smith EE (2010). Leukoaraiosis and stroke. Stroke.

[5] Mogi M, Horiuchi M (2011). Clinical Interaction between Brain and Kidney in Small Vessel Disease. Cardiol Res Pract.

[6] Horstmann S (2015). Prevalence of atrial fibrillation and association of previous antithrombotic treatment in patients with cerebral microbleeds. Eur J Neurol.

[7] Wada M (2008). Cerebral small vessel disease and chronic kidney disease (CKD): results of a cross-sectional study in community-based Japanese elderly. J Neurol Sci.

[8] Takahashi W, Tsukamoto Y, Takizawa S, Kawada S, Takagi S (2012). Relationship between chronic kidney disease and white matter hyperintensities on magnetic resonance imaging. J Stroke Cerebrovasc Dis.

[9] Khatri M (2007). Chronic Kidney Disease Is Associated With White Matter Hyperintensity Volume: The Northern Manhattan Study (NOMAS). Stroke.

[10] Akoudad S (2015). Kidney function and cerebral small vessel disease in the general population. Int J Stroke.

[11] Kim SH (2017). Kidney dysfunction and cerebral microbleeds in neurologically healthy adults. PLoS One.

[12] Levey AS (2006). Using standardized serum creatinine values in the modification of diet in renal disease study equation for estimating glomerular filtration rate. Annals of internal medicine.

[13] Levey AS (2003). National Kidney Foundation practice guidelines for chronic kidney disease: evaluation, classification, and stratification. Annals of internal medicine.

[14] Padwal R, Straus SE, McAlister FA (2001). Evidence based management of hypertension. Cardiovascular risk factors and their effects on the decision to treat hypertension: evidence based review. BMJ.

[15] Ueshima H (2008). Cardiovascular disease and risk factors in Asia: a selected review. Circulation.

[16] KDOQI. KDOQI Clinical Practice Guidelines and Clinical Practice Recommendations for Diabetes and Chronic Kidney Disease. *Am J Kidney Dis* **49**, S12–154, 10.1053/j.ajkd.2006.12.005 (2007). 10.1053/j.ajkd.2006.12.005 17276798

[17] Pantoni L (2010). Cerebral small vessel disease: from pathogenesis and clinical characteristics to therapeutic challenges. The Lancet Neurology.

[18] Hong KS (2013). Stroke statistics in Korea: part I. Epidemiology and risk factors: a report from the korean stroke society and clinical research center for stroke. Journal of stroke.

[19] Molino D, De Lucia D, Gaspare De Santo N (2006). Coagulation disorders in uremia. Semin Nephrol.

[20] Ambuhl PM, Wuthrich RP, Korte W, Schmid L, Krapf R (1997). Plasma hypercoagulability in haemodialysis patients: impact of dialysis and anticoagulation. Nephrol Dial Transplant.

[21] Sandsmark DK (2015). Proteinuria, but Not eGFR, Predicts Stroke Risk in Chronic Kidney Disease: Chronic Renal Insufficiency Cohort Study. Stroke.

[22] KDIGO. KDIGO 2012 Clinical Practice Guideline for the Evaluation and Management of Chronic Kidney Disease (2013). 10.1038/ki.2013.243 23989362

[23] James PA (2014). 2014 evidence-based guideline for the management of high blood pressure in adults: report from the panel members appointed to the Eighth Joint National Committee (JNC 8). Jama.

[24] Korean Centers for Disease Control. Korea Health Statistics 2013: Korea National Health and Nutrition Examination Survey (KNHANES VI-1) (2014).

