# Supplementary material for: Kidney Dysfunction Impact on White Matter Hyperintensity Volume in Neurologically Healthy Adults
Source: Sci Rep. 2019 Jun 13;9:8596. doi: 10.1038/s41598-019-45109-y (PMC6565737; doi:10.1038/s41598-019-45109-y)
Supplement: Supplementary file 1 — Baseline characteristics of Included and Excluded subjects based on the Urine Albumin-to-Creatinine Ratio [file 41598_2019_45109_MOESM1_ESM.docx]

**Article type:** Original article

**Kidney Dysfunction Impact on White Matter Hyperintensity Volume in Neurologically Healthy Adults**

**Short Title**: Kidney Dysfunction and WMH Volume

Sang Hyuck Kim, MD,^1*^ Jae Moon Yun, MD,^2*^ Su-Min Jeong, MD,^2^ Shinhye Kim, MD,^3^ Tae GonYoo, MD, MPH,^2^ Ji Eun Lee, MD,^2^ Jae-Sung Lim, MD,^4^ Han-Yeoung Jeong, MD,^5^ Ki-Woong Nam, MD,^5^ Hyung-Min Kwon, MD, PhD,^5,6§^ Jin-Ho Park, MD, PhD^2,7§^

* Sang Hyuck Kim and Jae Moon Yun contributed equally as first authors.

**Affiliations:**

1 Department of Family Medicine, Bumin Hospital, Seoul, Republic of Korea

2 Department of Family Medicine, Seoul National University Hospital, Seoul, Republic of Korea

3 Department of Family Medicine, Gangnam Severance Hospital, Seoul, Republic of Korea

4 Department of Neurology, Hallym University Sacred Heart Hospital, Anyang, Republic of Korea

5 Department of Neurology, Seoul National University-Seoul Municipal Government Boramae Medical Center, Seoul, Republic of Korea

6 Department of Neurology, Seoul National University College of Medicine, Seoul, Republic of Korea

7 Department of Family Medicine, Seoul National University College of Medicine, Seoul, Republic of Korea

**Corresponding Authors:**

§ Jin-Ho Park and Hyung-Min Kwon contributed equally as corresponding author

**Jin-Ho Park, MD, MPH, PhD**

Department of Family Medicine, Seoul National University College of Medicine

Department of Family Medicine, Seoul National University Hospital

101 Daehak-ro, Jongro-gu, Seoul 03080, Republic of Korea

Tel: +82.2-2072-0865, Fax: +82.2-766-3296

e-mail: kkolzzi0@gmail.com

**Hyung-Min Kwon, MD, PhD**

Department of Neurology, Seoul National University College of Medicine

Department of Neurology, Seoul National University-Seoul Municipal Government Boramae Medical Center, 20 Boramae-ro 5-gil, Dongjak-gu, Seoul 07061, Republic of Korea

Tel: +82.2-870-2475, Fax: +82.2-831-2826

e-mail: [hmkwon@snu.ac.kr](mailto:hmkwon@snu.ac.kr)

| **Supplementary Table 1. Baseline characteristics of Included and Excluded subjects based on the Urine Albumin-to-Creatinine Ratio** | | | | | |
| --- | --- | --- | --- | --- | --- |
|  | Included  (n = 2203) | | Excluded  (n = 869) | | *P*^‡^ |
| White Matter Hyperintensity Volume, cm^3^ | 2.7 | ± 6.0 | 2.7 | ± 7.5 | 0.998 |
|  |  |  |  |  |  |
| Age (year) | 56.9 | ± 8.3 | 58.3 | ± 8.8 | <0.001 |
| Male, n (%) | 1215 | (55.2) | 430 | (49.5) | 0.005 |
| Current Smoker, n (%) | 410 | (18.6) | 759 | (87.3) | <0.001 |
| Taking Anti-Hypertensive Drugs, n (%) | 551 | (25.0) | 242 | (27.9) | 0.106 |
| Taking Anti-Diabetic Drugs, n (%) | 154 | (7.0) | 74 | (8.5) | 0.146 |
| Taking Anti-Dyslipidemic Drugs, n (%) | 177 | (8.0) | 106 | (12.2) | <0.001 |
| Taking Anti-coagulation or Anti-Platelet Drugs*, n (%) | 224 | (10.2) | 135 | (15.5) | <0.001 |
| Body Mass Index - kg/m^2^ | 24.1 | ± 3.0 | 24.3 | ± 3.1 | 0.066 |
| Systolic Blood Pressure - mmHg | 126.6 | ± 15.8 | 125.2 | ± 14.8 | 0.030 |
| Diastolic Blood Pressure - mmHg | 76.3 | ± 10.6 | 75.6 | ± 10.6 | 0.104 |
| Creatinine - mg/dL | 0.92 | ± 0.21 | 0.86 | ± 0.22 | <0.001 |
| eGFR† - ml/min/1.73 m^2^ | 78.0 | ± 14.8 | 82.7 | ± 16.3 | <0.001 |
| Total Cholesterol - mg/dL | 200.7 | ± 35.6 | 198.3 | ± 37.2 | 0.099 |
| Low Density Lipoprotein - mg/dL | 128.1 | ± 34.6 | 124.3 | ± 35.1 | 0.010 |
| High Density Lipoprotein - mg/dL | 54.5 | ± 13.7 | 55.4 | ± 14.6 | 0.112 |
| Triglyceride - mg/dL | 119.6 | ± 70.0 | 118.0 | ± 76.9 | 0.572 |
| Fasting Blood Glucose - mg/dL | 96.2 | ± 22.3 | 99.0 | ± 25.0 | 0.002 |
| Hemoglobin A1c | 5.92 | ± 0.78 | 5.85 | ± 0.81 | 0.029 |
| eGFR, estimated glomerular filtration rate  Values are shown as means ± standard deviation.  * Subjects taking aspirin, clopidogrel, warfarin, or other antiplatelet drugs  † Calculated using the Modification of Diet in Renal Disease formula  ‡ *P* values were calculated by chi-square test for categorical variables or t-test for continuous variables | | | | | |
